# Exposure Assessment to Radiofrequency Electromagnetic Fields in Occupational Military Scenarios: A Review

**DOI:** 10.3390/ijerph19020920

**Published:** 2022-01-14

**Authors:** Silvia Gallucci, Serena Fiocchi, Marta Bonato, Emma Chiaramello, Gabriella Tognola, Marta Parazzini

**Affiliations:** 1 Institute of Electronics, Computer and Telecommunication Engineering (IEIIT), CNR, 20133 Milan, Italy; serena.fiocchi@ieiit.cnr.it (S.F.); marta.bonato@ieiit.cnr.it (M.B.); emma.chiaramello@ieiit.cnr.it (E.C.); gabriella.tognola@ieiit.cnr.it (G.T.); marta.parazzini@ieiit.cnr.it (M.P.); 2 Department of Electronics, Information and Bioengineering (DEIB), Politecnico di Milano, 20133 Milan, Italy

**Keywords:** EM fields, occupational exposure, military environment, military devices

## Abstract

(1) Background: Radiofrequency radiations are used in most devices in current use and, consequently, the assessment of the human exposure to the radiofrequency radiations has become an issue of strong interest. Even if in the military field there is wide use of radiofrequency devices, a clear picture on the exposure assessment to the electromagnetic field of the human beings in the military scenario is still missing. (2) Methods: a review of the scientific literature regarding the assessment of the exposure of the military personnel to the RF specific to the military environment, was performed. (3) Results: the review has been performed grouping the scientific literature by the typology of military devices to which the military personnel can be exposed to. The military devices have been classified in four main classes, according to their intended use: communication devices, localization/surveillance devices, jammers and EM directed-energy weapons. (4) Discussion and Conclusions: The review showed that in the exposure conditions here evaluated, there were only occasional situations of overexposure, whereas in the majority of the conditions the exposure was below the worker exposure limits. Nevertheless, the limited number of studies and the lack of exposure assessment studies for some devices prevent us to draw definitive conclusions and encourage further studies on military exposure assessment.

## 1. Introduction

The spread of the technologies based on electromagnetic fields (EMF) is constantly increasing and it corresponds to the huge development and diffusion of devices using them. In particular, in both civil and military environments, the most used electromagnetic radiation lies in the radiofrequency (RF) range. With the increasing use of these technologies, many questions have been raised about the possible biological effects associated with exposure to RF radiation.

The interaction between electromagnetic fields and health has been deeply investigated in the last 30–40 years particularly for the devices typically used in the civil environment [1,2,3,4,5], whereas for the devices used in the military environment more investigation is needed. Indeed, technological breakthroughs have been leading to a progressively increasing exposure of military personnel to high-intensity radiofrequency radiation [6]. Moreover, even if the military personnel need the regulations limiting the exposure to prevent harmful effects, at the same time the usage of RF devices should not be overly limited. Moreover, in a typical military environment, there is more than one source emitting RF radiations simultaneously, and besides, these sources are often characteristic only of military settings.

In the scientific literature, the issue of the exposure of the military personnel to the radiofrequency fields and its interaction with health has been addressed from several points of view, such as performing: (i) occupational epidemiological studies focusing on the exposure of specific military device (see for example [7]); (ii) in-vitro or in-vivo studies in which biological samples or animals are irradiated inside an exposure system (i.e., propagating systems, radiating systems) to EMF at specific frequencies and the related biological responses are evaluated (see for example [8]); (iii) exposure assessment by experimental or numerical (or in silico) dosimetry studies (see for example [9]). However, the number of studies related to exposure to EMF in the military environment is lower than in the civilian environment and there is still no clear picture on the human exposure to RF EMF in the military scenario. As example, there are studies that suggest a cause-effect relationship between several RF military sources (i.e., radar for surveillance, radio communications) and the hematolymphatic (HL) cancers in occupational groups [10] and a higher incidence of all malignancies in RF/microwave (MW)-exposed military personnel with respect to unexposed personnel of the same socio-economic condition and working conditions [11]. On the other hand, there are also epidemiological studies suggesting the lack of relationship between the exposure to radar radiations and the occurrence of diseases related to it; this is the case of Dabouis et al. [7] and Degrave et al. [12]. Dabouis et al. [7] performed a retrospective cohort study on 39,850 military workers, belonged to the French Navy, observed during a period coming from 1975 to 1995. The cohort was divided into two groups: a control group (n = 18,310) and a radar exposed group (n = 21,540). The mortality incidence rate ratio (IRR) and the cancer death incidence rate resulted not significantly different between the exposed and control group. In the work by Degrave et al. [12] a statistical approach was performed for calculating the mortality ratio in a group of 31,616 military workers and a control group of 18,631. The results have shown there was no increase in mortality in Belgian militaries compared to the civilians. Nevertheless, in these typologies of studies, there is often neither a clear distinction about the typology of the sources nor a detailed description of the exposure conditions and exposure characterization. Consequently, a review of the studies carried out in this context is needed, especially as regards the quantification of EMF exposure in military occupational scenarios.

In this document, we aimed to fill this gap of knowledge reviewing the scientific literature regarding the assessment of the exposure of the military personnel to the RF specific to the military environment. To this end, the review has been performed grouping the scientific literature according to the typology of military devices to which the military personnel can be exposed to. The military devices have been classified in four main classes, according to their intended use: communication devices, localization/surveillance devices, jammers, and EM directed-energy weapons [10,13]. Moreover, we chose to narrow the frequency range to frequencies ranging from MHz to hundreds of GHz since the frequencies of most of the military devices belonging to the previously identified classes belong to this range.

## 2. Materials and Methods

A systematic literature search of the results was carried out on scientific literature, more specifically on literature published in Scopus and Google Scholar databases; scientific material research of the peer-reviewed articles published in the English language was completed by searching for military datasheets in order to have more knowledge about devices’ specifications.

Some of the most used terms to articulate the search have been:

- Military electromagnetic field;
- Military electromagnetic field exposure;
- Electromagnetic military devices;
- Occupational electromagnetic exposure assessment;
- Electromagnetic exposure in military environments.

The search expressions were chosen as a combination of various terms that describe the military environment (terrestrial, aerial, etc.), the exposure characteristics (frequency range, pulsed signal or not, etc.), and the typology of military devices (communication devices, localization/surveillance devices, jammers, and EM directed-energy weapons).

Firstly, the information extracted from each article has regarded the typology of military device analyzed in the study and the typology of the study (environmental or personal measurements, numerical dosimetry, etc.). Both of these information findings were crucial to categorize the found articles and to understand which type of data and results could be extracted. Particular attention has been focused on the exposure conditions, extracting from each selected article all the values assumed by the parameters describing the electromagnetic exposure (such as for example the frequency, the source’s input power, specific absorption rate level, etc.). In addition to the parameter itself, in all the articles where it was possible, information was also extracted on how the exposure levels had been compared with the limits or recommendations considered in the article itself (such as ICNIRP Guidelines 1998 [14], Directive 2013/35/EU [15], FCC Guidelines [16], etc.) and if these limits had been respected or exceeded.

Consequently, the peer-reviewed articles were selected according to the following inclusion criteria:

1. They included any typology of study related to the exposure assessment in military environment to the EMF in the frequency range between 1.5 MHz to 300 GHz;
2. They included devices currently used in the armed forces;
3. They included information about the exposure conditions;
4. They were published in the last 25 years.

## 3. Results

In this section, the results are organized in four paragraphs, each one for the different typology of military devices identified. The first one includes the communication technologies, the second paragraph is dedicated to the localization/surveillance devices, the jammer systems are included in the third part and, finally, the last paragraph is devoted to the EM directed-energy weapons. Table 1 lists the identified devices grouped according to the above subdivision. Each following section will be introduced by a short description of the military device analyzed in the cited studies.

### 3.1. Communication Devices

The term “communication” means the transmission of information between two entities. In a military environment, the transmitted information could consist, for example, both in audio/video messages and/or in the position of the soldier on a battlefield. In this class of devices, we have focused on four technologies widely used in the military environment: GSM technologies, radio transceivers, software-defined radios (SDR), and wearable devices.

The global system for mobile communications (GSM) is the second-generation standard for cellular communications; in detail, the GSM technology is one of the wireless applications in the communication field [17]. Until a decade ago, GSM technology was the most widespread cellular standard, with its frequency bands of 900 MHz or 1800 MHz [18]. The GSM is a civil communication standard, but its features are helpful also in the military environment, such as personal mobility, wireless access, the cellular radio network architecture, and so on. Therefore, a great deal of effort has been made to ensure that this technology was reconverted into the military sector so as to exploit its advantages [19].

The telecommunication systems in the military field also use the radio-band in the frequency range coming from 1.5 MHz to 90 MHz, in the wide band of VHF (very-high frequency) and to cover a wide range of action to communicate over a great distance, the input power imposed to the system ranges between 0.5 W and 10 W [20]. Often, the entire radio station, during the use, is placed on the backpack of the soldier and often the radio station is equipped with a rod antenna [21].

An enabling technology for developing military communications systems is the software-defined radio (SDR). SDR is a technology where several of the typical components such as filters, mixers, modulators, and demodulators of communications systems are implemented in software. SDR can be defined as the evolution of GSM technology in the military environment. The SDR is very advantageous for its reconfigurability, the capability of multimode operations [22], and therefore, allows much more flexibility than the classical radio transmitters. Their main advantages are that frequency and waveform can be briefly defined and it allows encryption and frequency hopping. With respect to the GSM technology, the SDR systems have a wider frequency range coming from 20 MHz to 6 GHz [23,24,25,26].

One of the most recent fields of military application on communication devices regards wearable devices. This type of technology is called “wearable” because it is positioned on the body of the user in order to reduce the obstacle of the antenna to the movements of the soldier and to allow hands-free operations. Furthermore, the antenna for the wearable devices must be miniaturized because the own sizes shall be compatible with the portability and the small footprint. One of the aims of these devices is to communicate the position of the soldier on a battlefield or in a mission. These devices must be comfortable, and the used antennas must be easily integrated into the soldier uniform [27]. Although there is no standard position of the antenna on the individual equipment, the antenna is often positioned on the helmet or on the jacket of the soldier [28]. As in the case of the SDR, the frequency band is wide-ranging from 30 MHz to 6 GHz [20,29,30].

In Table 2, the frequency ranges for the above communication devices are summarized.

Considering the exposure assessment of the military personnel to the EMF radiations emitted by the communication devices, the only studies in the literature specifically focused on military application concern some radio communication units and the wearable devices. For the other technologies, especially for the GSM system, it can be possible to refer to the studies and the findings obtained considering their civil applications, e.g., [1,2,3,4]. Therefore, these last communication devices will not be further analyzed in the present paper.

Karpowicz et al. [21] studied the exposure assessment to portable radio communication units (known as a radiophones) equipped with rod antenna and operating at a frequency of approximately 27 MHz used by military (and civilian) services. In particular, they performed measurements of limb induced current at the ankle of 24 volunteers standing near a radio communication rod antenna at two different distances and mapped the electric-field spatial distribution near the antenna itself. Moreover, in silico simulations mimicking the same exposure scenario of the experimental study were performed in four numerical body phantoms (two males and two females). The chosen scenario was representative of real-life exposure condition such as the exposure of soldiers staying outside a vehicle equipped with the radiophone. Results show compliance with the European Directive on worker’s exposure limits [15]. Kieliszek et al. [31] performed a study assessing the exposure due to the use of a portable radio. Specifically, they performed the measurements of the electric field distribution at 10 cm from the antenna of a typical commercial portable radio working at 30, 55 and 80 MHz for three typical radiophone powers (0.1, 1, 5 W). Using the same radiophone operating parameters as for the EMF distribution analysis, the limb contact current in the forearm of volunteers was measured during a conversation of the radiophone users. Moreover, specific absorption rate (SAR) estimation was performed considering a radiophone powered with 5 W. The radiophone model was placed in the hand of the operator’s body model at 10 cm from the head. The assessment indicated a large spatial variability of the electric field strength around the devices. The head of the operator and the hand holding the radio were often exposed to EMF levels which exceeded the established limits, particularly when the radiophone operated at 5 W. However, the calculated SAR values always ranged within the permitted limits. Specifically, SAR averaged over the whole body was below 0.017 W/kg, which was no more than 5% of the limit; the maximum local SAR_10g_ averaged on head and trunk was equal to 0.29 W/kg, while the maximum local SAR_10g_ averaged in the limb was 9.03 W/kg, both values well below the limits of the European Directive on worker’s exposure [15].

Sobiech et al. [13] measured the electric field of three groups of radio transceivers used by the Polish Army: portable and hand-held radios, manpack radios and high frequency/very high frequency (HF/VHF) band devices installed in vehicles. Portable radios (working between 42 to 50 MHz at 0.5–5 W of transmitted power) emitted an electric field strength between 20–80 V/m close to a human head (the minimum distance between the probe and the antenna was 15 cm). The manpack radio operator exposure depends on the placement of the equipment (working between 30–90 MHz, at 5–20 W of transmitted power). When the radio is transported on the shoulders then the antenna is placed in proximity (about 30 cm) to the human head, the operator’s exposure was 60–120 V/m, exceeding the limits imposed for this frequency range by the European Directive [16]. Inside vehicles with HF/VHF band radios (working between 1.5 to 90 MHz, at 50–1000 W of transmitted power), the electric field strength was between 7–30 V/m.

Paljanos et al. [32] estimated the exposure in the immediate vicinity of a portable radio communication transceiver working in the frequency band 30–108 MHz by both measurements and computational methods. Measurements were made in situ using a broadband personal exposimeter equipped with two isotropic probes for both electric and magnetic components of the field. Simulations were performed in a homogeneous human head posed at 20 cm of distance from the source. Exposure levels at 30 MHz exceeded the exposure limits at 20 cm away from radiating source whereas for the other two considered frequencies compliance with limits was assured. Moreover, SAR_10g_ values were always far below the exposure limit not revealing any condition of overexposure (EU Directive [15] and ICNIRP 1998 [14]).

As for wearable devices, in recent years, they have also become the subject of investigation from the point of view of the interactions between the EMF radiations emitted by them and human tissues since the human body is very close to the RF source. For this purpose, in the literature some studies have been focused on the exposure assessment in military personnel wearing wearable devices by means of computational electromagnetic techniques, performing in-silico analysis. In the first one [9], a bent antenna posed at 10 mm from the specific anthropomorphic mannequin (SAM) head phantom was simulated via Sim4Life software (ZMT Zurich MedTech AG, www.zmt.swiss, accessed on 14 November 2021), mimicking a wearable antenna integrated into a military beret. The peak SAR_1g_ resulted equal to 0.0252 W/kg and 0.175 W/kg for f = 1.575 GHz and 915 MHz, respectively, both at 1 W input power. Poonkuzhali et al. [33] modelled an antenna on a human arm operating at 1 W input power at 450 MHz and found a peak SAR equal to 0.1427 W/kg. An E-shaped patch wearable textile antenna was considered in [34] and it was positioned on a torso model; in this study, the peak SAR_1g_ for f = 1.85 GHz and f = 2.45 GHz, with the input power of 1 W, resulted equal to 0.54 W/kg and 0.35 W/kg, respectively. On the other hand, there are studies in which the values of the SAR are higher than the previously presented results, e.g., Chahat et al. [35] shows peaks SAR_1g_ with input power of 1 W in a homogeneous phantom equal to 48 W/kg (f = 2.45 GHz), 50.9 W/kg (f = 2.59 GHz) and 67.4 W/kg (f = 5.5 GHz) when the antenna was placed on the body. Furthermore, the results demonstrated that the peaks SAR_1g_ were significantly reduced when the antenna was integrated with an electromagnetic band gap (EGB). Indeed, the EBG structure reduced the peaks SAR_1g_ at 1.7 W/kg, 2.3 W/kg and 1.0 W/kg at 2.45 GHz, 2.59 GHz, and 5.5 GHz, respectively. Furthermore, Chahat et al. [36] performed another study in which they modelled a wearable dual-band textile antenna working at f = 2.4 GHz and 5.5 GHz posed at 1 mm from the chest of four anatomical whole-body model of the virtual family [37]: the peak SAR_1g_ obtained with an input power of 1 W ranged from 34.2 W/kg in the case of the female child (11 years-old) to 18.7 W/kg in the case of the adult female for the antenna operating at 2.4 GHz, and from 14.8 W/kg in the case of the female child to 16.4 W/kg in the case of male child (6 years old) for the antenna at 5.5 GHz. Michishita and Morishita [38] developed a helmet antenna working at 150 MHz to achieve hands-free operations and simulated the SAR_10g_ distributions in a human head model. The unwanted radiation toward the human head resulted suppressed, and the maximum SAR_10g_ value resulted equal to 0.67 W/kg, which is lower than the safety limit. Nasim and Kim [39] investigated the EMF exposure effect from on-body wearable devices at 2.4 GHz, and their results suggested that SAR does not exceed the exposure guidelines (ICNIRP 1998 [14] and FCC guidelines [16]).

### 3.2. Localization/Surveillance Devices (Radar)

In a military environment, the protection of the headquarter is essential whereby there is the need for a system for surveillance of the surrounding area and detection and tracking of potential threats on the ground and in the air, as well as monitoring of movements of own forces. This task is performed by radar as well as with electro-optical sensors. Radar technology is a system that detects the position or the velocity of an object by using radio waves; in particular, radars are systems able to detect both fixed and moving objects by means of microwave radiations and therefore, depending on the tasks, different frequencies in the microwave spectrum are used [40].

The frequency of the radar systems ranges from 1 GHz to 300 GHz [7,41,42] and this variability of the frequencies depends on the application (i.e., control radars, weather radar, etc.). Indeed, the radar spectrum can be divided into 11 parts, where each part identifies a band: L-, S-, C-, X-, Ku-, K-, Ka-, V-, W-band [13]. For example, in the L-Band (1–2 GHz) the 3D radar operates; this type of radar can detect and track targets in terms of position (range, azimuth, and elevation) at ranges up to 400 km due to the ability to rotate the two-dimensional antenna [13]. While the maximum range is decreasing with increasing transmitted frequency (while the resolvable target size decreases with increasing frequency), 3D target detection and tracking are possible at all radar frequencies, as this only depends on the system design.

The frequency range of the localization devices is reported in Table 3.

Because of the huge spread of the radar systems due to their many different applications, this type of localization system has sparked much interest in terms of exposure assessment of the workers.

Singh and colleagues [42,43] performed a study on 166 active soldiers of the Indian Army who were categorized in three different groups according to their exposure to electromagnetic radiations emitted from radar: group I (n = 40, X-band radar frequency range 8–12 GHz), group II (n = 58, Ku-band radar frequency range 12.5–18 GHz), and control group (n = 68). Besides physiological parameters, electromagnetic fields’ levels were measured at different locations (inside radar cabin, at the top front of radar vehicle, and occupational spots within the 50 m range where personnel were supposed to be present during their duty). Measurements represent instantaneous readings taken at different locations of the personnel’s workstation/environment in the course of the daily work schedule during radar operations and maintenance. Power density measurements inside and outside the radar cabin at various occupational locations ranged from 0.24 to 0.77 W/m^2^ in case of Exposure Group I, whereas varied from 0.1 to 15.6 W/m^2^ in case of Exposure Group II. All these values were below the exposure limit for occupational exposure [5,14].

Sobiech et al. [13] shows that inside the radar cabin the electric field strength RMS value averaged over the pulse repetition period ranged from 9 to 20 V/m and, in the proximity of the antenna unit of the surveillance radar, the electric field strength RMS value averaged over the pulse repetition period and antenna rotation time was equal to about 30 V/m. All these levels were below the limits established in the EU Directive [15]. Similarly, in the Danulescu’s study [44] the average power density at radar workplaces was measured. At frequencies around 2–6 GHz, the average power densities were equal to 0.4–5 W/m^2^ (corresponding to 12–43 V/m calculated from the plane wave condition), and at frequencies smaller than 2 GHz equal to 3–10 W/m^2^ (33–61 V/m).

Sobiech et al. [13] shows also that personnel on ships were not exposed to the EMF emitted by their own radar systems. Similar conclusions are also obtained in Garaj-Vrhovac et al.’s study, [45] where the EMF strength was measured at assigned marine radar frequencies (3 GHz, 5.5 GHz and 9.4 GHz) working with peak power of 50–60 kW. The power density averaged over the pulse repetition period and the radar antenna rotation period was no more than 0.0002 W/m^2^ (0.3 V/m) at the radar operator workplace and 0.004 W/m^2^ (1.2 V/m) in the day rest area and sleeping quarters. In line with these results, Dabouis et al. [7] estimated the power density in different areas occupied by the military population belonged to the French Navy surface vessels. Electric field measurements were taken in locations where radar exposure level was supposed to be maximum according to numerical simulations. The data were derived from 50 measurements of the electric field taken at different points on the deck (identified as exposed locations) and 10 locations under the deck (identified as control locations), considering the radar frequencies in the L (1300–1375 MHz), S (2900–3200 MHz) and X (9380–9450 MHz) bands. All exposure levels measured on the vessel were below the limit values, recommended by the ICNIRP for occupational exposure safety (ICNIRP 1998 [14]). Hjollund and Bonte [46] indicated the exposure of Danish military personnel operating mobile ground-to-air missile units that used several microwaves emitting radar systems. In this study, the maximal mean exposure was estimated to be 0.1 W/m^2^ (6 V/m). Short term exposures of approximately 10 W/m^2^ (61 V/m) might occasionally occur.

### 3.3. Jammers

The jammer is a device able to interrupt the communication channel [47] and block the signal by emitting a series of electromagnetic pulses [48] at the same frequencies of the signal to hinder. In a military environment, this ability is essential to interrupt enemy communications. A characteristic aspect is the signal power because, for being effective, the jammer power signal must be equal or higher than the signal power at the receiver, at least one order of magnitude [49], so that the two signals can collide and cancel each other [50]. Another equally important application of jammers, also called electronic counter measures (ECM) [51] is the interruption of enemy radar surveillance by several specific jamming techniques through which the enemy loses the ability to detect, track and visualize objects belonging to the own and friendly forces.

In order to fulfill different military capabilities needs, jammers can be classified as mobile and stationary. Mobile includes man-portable, land vehicle portable, and airborne [52]. The man-portable jammer is installed inside a case so that use in adverse (environmental, weather-related, etc.) conditions does not compromise its functionalities and the frequencies range from approximately 100 MHz to 2.7 GHz. Its purpose is to protect against improvised explosive devices (IEDs) and disrupt enemy communications. The land vehicle jammers are again designed for vehicle passengers’ protection against IEDs and to disrupt enemy communication (analog to the portable jammers). For this type of jammer, the frequencies come from 20 MHz to 6 GHz. Another mobile type of jammer is installed in military aircraft. Airborne jammers can be used both for disruption of enemy communication networks and for the interference of enemy radars in the air or on the ground (suppression of enemy air defence, SEAD). Finally, the stationary jammer is a solution for disrupting unwanted communications from handheld devices and unmanned aerial vehicles (UAVs). This type of jammer is a high-power solution for protecting, for example, against terrorist attacks or against espionage. For the stationary jammer, the frequencies range from 900 MHz to 6 GHz. In Table 4 the different typologies of jammers are reported with their frequency ranges.

Regarding the exposure of the military personnel, the jammer is a relevant device from the point of view of the interactions between the RF radiations emitted by the jammer’s antenna and the human tissues because the device with its antenna and the soldier are very close, such as in the case of the man-portable jammer in which the source is brought by the soldier on his shoulders.

Yahya et al. [53] have performed in-silico simulations: a jammer with three antennas tuned on three different frequencies (i.e., 900 MHz, 1800 MHz, and 2100 MHz) has been tested with three different human models: Eartha, Ella (both from the Virtual Family [37]) and the visible human (VH) phantom, at four different distances between the jammer and the human model (20 cm, 50 cm, 100 cm, and 200 cm). By means of computational techniques, the SAR values normalized to an input power of 1 W have been calculated and the results showed that, at the shortest distance, the highest value of the whole-body averaged SAR was 6.90 × 10^−3^ W/kg, found in the Eartha model, and at the greatest distance the whole-body averaged SAR decreased to 4.98 × 10^−5^ W/kg in the VH model. However, none of these estimated values exceeds the allowed limit of the SAR (ICNIRP 1998 [14], FCC Guidelines [16]).

### 3.4. EM Directed-Energy Weapons

In recent years, there has been a strong development of research in non-lethal weapons matter. This choice is a consequence of collateral civilian damages in many military operations [54]. The directed-energy weapons (DEW) belong to the class of non-lethal weapons and their operation is based on a focused energy beam that destroys the circuitry in any electronic device without special military hardening.

DEWs are based on high-power microwave (HPM) technology. This non-lethal technology is based on the emission of very intense short electromagnetic pulses to such an extent that—among military equipment—electronic circuits in communications in different bands (according to the design of the DEW) are at risk to be destroyed or at least their function degraded if they are not sufficiently hardened. Much more at risk, however, is civilian commercial electronic equipment, which is not hardened against such attack and those will be surely destroyed [55]. The typical HPM weapon is composed of a pulsed power unit, an antenna with high gain, and a microwave source, and the output frequency of these devices typically ranges from 1 GHz to 100 GHz [56,57].

A particular branch of non-lethal weapon research regards the ability to immobilize a moving vehicle. The operating principle is based on the use of an HPM source interfering with the vehicle electronics [58]. The effect of this type of weapon in the frequency range from 200 MHz to 5 GHz is the damage only to the electronic devices [59]. More specifically, this weapon has the aim to stop the engine of stationary or moving vehicles and to protect the convoys [60]. The system stopping the vehicles can be both mounted on a vehicle and portable using a battery system [61].

The abovementioned devices are summarized in Table 5.

In terms of exposure of the military personnel to the radiations emitted by the abovementioned systems, the only typology of device that is consistent with the current review is the car stopper device in the matter of accidental exposure of the workers nearby the car stopper device. However, until now, in the literature, no studies about the assessment of the RF exposure of the military personnel in this specific scenario have been found.

## 4. Discussion

The present study was performed to review the scientific literature regarding the EMF exposure assessment on occupational/military settings, with particular focus on RF exposure. The major uses of RF in the military environment are communication devices, localization/surveillance devices, jammers, and EM directed-energy weapons. Therefore, the literature has been previously grouped according to the intended use of the military device, showing a wide variability of the exposure conditions of the military personnel.

**Figure 1:**
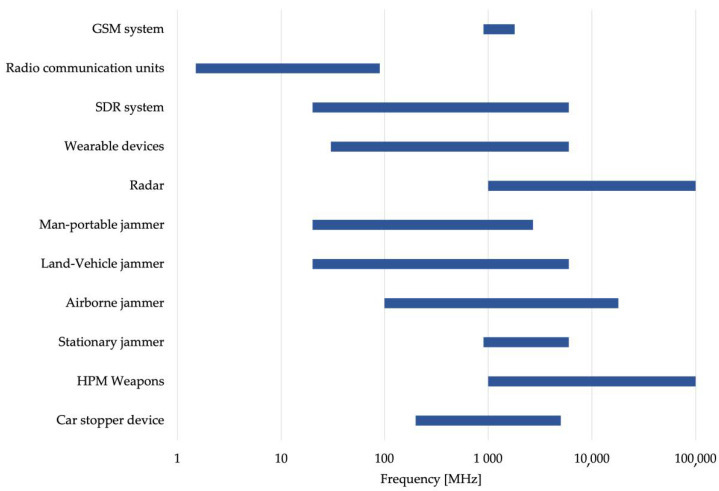
Distributions of the frequency ranges in the radiofrequency spectrum.

However, it could be useful to organize the studied devices also according to their working frequency, showing how these systems fit into the radiofrequency spectrum (Figure 1) and to differentiate the sources according to the way of the use of each device (Table 6), considering if they are used near or far from the human body. All these aspects could indeed influence the exposure conditions, leading for example to exposures of the whole body or mainly of some parts of the body of operators and/or bystanders during a normal operation mode of the device.

As shown in Figure 1, most of the typology of devices can work in a frequency band up to 6 GHz, whereas only a few can reach higher frequencies (up to 10 GHz).

Table 6 shows a possible classification of the devices here analyzed according to their position relative to the military worker, with their most typical configuration of use (i.e., near or far from the user). The devices used at a limited distance between the source and the human being are the communication devices and the most used are the jammers, i.e., man-portable and land vehicle. The distance between the source and the human being is another variable that greatly influences the EMF exposure, typically reducing the exposure with increasing distance. This is true also in the case of the exposure of military personnel.

In general, if we compare the data here reviewed with the limits established by the European Directive 2013/35/EU [15], it appears that in the exposure conditions here evaluated, there were only occasional situations of overexposure, whereas in most of the conditions, the exposure was below the worker exposure limits. The choice to use the European Directive 2013/35/EU [15] as a reference lies in its mandatory nature for all the European countries. By making this comparison, it was highlighted that in almost all cases the limits were respected, in terms of exposure limits values (ELVs) and/or in terms of action levels (ALs). This is truer for the radiocommunication devices for which both experimental and dosimetric studies were performed [20,21], and localization/surveillance devices (radar), for which exposure measurement campaigns were performed in areas occupied by the military personnel [7,31,42,43,44,45]. Conversely, exposure assessment of the EMF generated by jammer devices and wearable devices is still limited or, as in case of the EM directed-energy weapons, completely lacking. For the former, particularly, since they make use of omnidirectional antennas often positioned very close to the body and operating at power levels up to hundreds of watts, studies aimed to circumscribe eventual overexposure scenarios are advisable. Moreover, the typical use of all these devices implies a large variability in the plausible exposure scenarios which, in turn, shall severely impact on the variability of the exposure levels. Sources of this uncertainty are, for example, the relative position between the EMF sources and the exposed subject, the different power contribution for each operating frequency, the timing of the power signal, the different anatomical characteristics of the exposed subject.

For all these reasons, the findings here summarized are not conclusive, and it is recommended to conduct further studies on military exposure assessment to these specific military devices.

In this context, it is important also to consider that, beyond all the radiofrequency sources here considered, in the future also the 5G technology will be included. In fact, when a soldier is on mission, he will need to communicate accurately to the headquarter his position, the images of the environment and other strategic data [62]. This amount of information is impossible to transmit with the current technologies whereas it will be possible to use the 5G technology. The introduction of this innovation will complicate even more the current EMF exposure scenario, increasing its variability and uncertainty, due to the involved innovation technologies (i.e., the use of mm-wave working frequencies, of MIMO antenna, of 3D beamforming techniques). All these aspects are not yet been studied in the military environment and it is therefore necessary to conduct promptly an exposure assessment, considering the new antenna technologies and frequencies involved.

In conclusion, the limited number of studies currently present in the literature and both the current and future multitude of exposure scenarios point out that there is a real need to increase EMF exposure assessment studies in military working conditions.

## 5. Conclusions

In conclusion, this study provides an overview of the current used military devices and of the exposure studies that have been performed to characterize the exposure environment in the military scenario with the aim to analyze the state of the art regarding the interaction between the soldier and the RF-EMFs emitted by military devices. The results include four different typologies of systems, each one for different intended use: communication, localization, jamming, and electronic weaponry.

The reviewed studies about the assessment of the human exposure to the radiation emitted by the above mentioned military devices suggest that in the exposure conditions here evaluated, there were only occasional situations of overexposure, but the large number of variables involved in the description of these scenarios, the wide heterogeneity of the exposure conditions and the absence of exposure evaluation for some specific devices make further studies necessary, also considering upcoming use of other technologies, such as 5G, which will dramatically change the exposure conditions also in military environment, both in terms of the time and conditions exposure and in terms of intrinsic sources characteristics, such as the used frequency ranges.

## Figures and Tables

**Table 1:** Military devices grouped by intended use.

Destination of Use	Devices
Communication devices	GSM systems
	Radio communication units and SDR systems
	Wearable devices
Localization/Surveillance devices	Radar
Jammers	Man-portable jammers
	Land vehicle jammers
	Airborne jammers
	Stationary jammers
EM Directed-Energy Weapons	HPM—High-power microwave weapons
	Car stopper devices

**Table 2:** Communication devices and relative frequency ranges.

Devices	Frequency Range
GSM systems	900 MHz–1.8 GHz
Radio Communication Units	1.5 MHz–90 MHz
SDR systems	20 MHz–6 GHz
Wearable devices	30 MHz–6 GHz

**Table 3:** Localization/surveillance devices and relative frequency ranges.

Device	Frequency Range
Radar	1–300 GHz

**Table 4:** Jammers and relative frequency ranges.

Device	Frequency Range
Man-portable jammers	20 MHz–2.7 GHz
Land vehicle jammers	20 MHz–6 GHz
Airborne jammers	100 MHz–18 GHz
Stationary jammers	900 MHz–6 GHz

**Table 5:** EM directed-energy weapons and relative frequency ranges.

Device	Frequency Range
HPM—High-power microwave weapons	1–100 GHz
Car stopper devices	200 MHz–5 GHz

**Table 6:** Devices with their configuration.

Destination of Use	Devices	Configuration
Communication devices	GSM systems	Near the user
	Radio communication units	Near the user
	SDR systems	Near the user
	Wearable devices	Near the user
Localization/surveillance devices	Radar	Far from the user
Jammers	Man-portable jammers	Near the user
	Land vehicle jammers	Near the user
	Airborne jammers	Far from the user
	Stationary jammers	Far from the user
EM Directed-Energy Weapons	HPM—high-power microwave weapons	Far from the user
	Car stopper devices	Far from the user
